# Beyond the Nut: *Pistacia* Leaves as Natural Food Preservatives

**DOI:** 10.3390/foods13193138

**Published:** 2024-09-30

**Authors:** Daniela Batovska, Moshe Inbar

**Affiliations:** 1 Institute of Chemical Engineering, Bulgarian Academy of Sciences, 1113 Sofia, Bulgaria; 2 Department of Evolutionary & Environmental Biology, University of Haifa, Haifa 3498838, Israel; minbar@research.haifa.ac.il

**Keywords:** antioxidant, antimicrobial, bioactive compounds, food applications, food preservation, health benefits, *Pistacia* spp., *Pistacia vera*, preservatives, valorization

## Abstract

The pistachio tree (*Pistacia vera*) is globally renowned for its nutritious nuts, while its leaves remain an underutilized source of chemicals with significant potential value as food preservatives. Similar value may be found in the leaves of other wild *Pistacia* species common in Central Asia, the Levant, and around the Mediterranean. Some species’ leaves have been used as natural preservatives, demonstrating their effectiveness and highlighting their rich bioactive components. This review investigates the antioxidant and antimicrobial properties of *Pistacia* leaves, comparing both cultivated and wild species. A comprehensive search was performed across several scientific databases, including PubMed, Scopus, Web of Science, and Google Scholar, utilizing a combination of keywords related to *Pistacia* species and their bioactive compounds. The inclusion criteria focused on articles published in English from 2017 till the end of June 2024, analyzing the antioxidant and antimicrobial activities of *Pistacia* leaves and employing relevant extraction methods. A total of 71 literature sources were included, covering species such as *P. vera*, *P. atlantica*, *P. terebinthus*, and others sourced from countries such as Iran, Turkey, and Italy. This review found that *Pistacia* leaves are rich in polyphenolic compounds and exhibit robust antioxidant and antimicrobial properties, with certain wild species outperforming *P. vera*, suggesting species-specific traits that enhance their preservative potential. The major findings indicate that extracts from wild species exhibit superior bioactivity, which could be harnessed for food preservation. These insights underscore the promising role of *Pistacia* leaves as natural food preservatives, with further research needed to address challenges in extraction and application. Exploring their synergistic effects with other preservatives could lead to innovative solutions in food preservation while fostering local economic growth.

## 1. Introduction

The genus *Pistacia* L. (Anacardiaceae) consists of about fifteen species of trees and shrubs [1]. The plants are dioecious, mostly deciduous with pinnately compound leaves. *Pistacia* has a wide disjunct distribution in the northern hemisphere, primarily in the Old World, and two species in North America [2]. In many areas, especially in West and Central Asia and around the Mediterranean these plants are dominant and deeply rooted in the culture and tradition of local communities that extensively used them [3].

**Figure 1:**
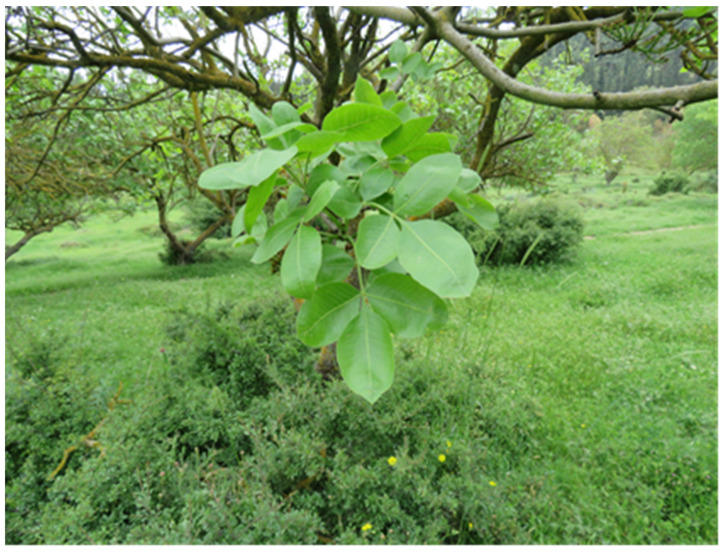
Young leaves of *Pistacia vera*.

**Figure 2:**
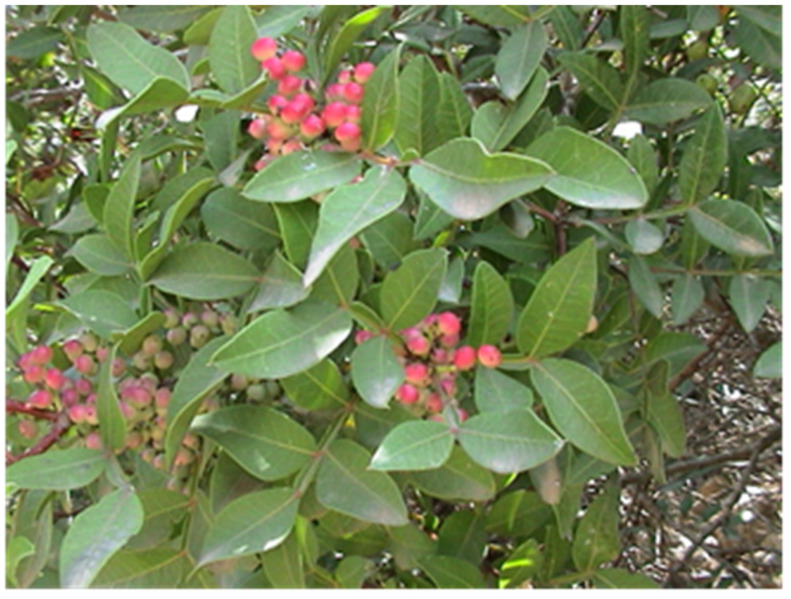
Leaves and fruits of the evergreen shrub *Pistacia lentiscus*.

*Pistacia vera* (Figure 1) and *P. lentiscus* var. chia are the only domesticated species in the genus, both of substantial economic value. While *P. vera* is globally recognized for its nutritious and flavorful nuts, *P. lentiscus* var. chia is particularly important in the production of mastic [4,5,6,7]. Their often-neglected leaves are rich in bioactive ingredients such as essential oils (EOs), terpenoids, and polyphenolic compounds. These compounds offer significant health advantages and have promising potential for applications in food preservation [7,8,9,10,11]. For instance, extracts from *P. vera* leaves, known for their strong antioxidant and antimicrobial properties, have been successfully utilized as bakery additives to improve bread quality and extend its shelf life [8]. Similarly, extracts from *P. lentiscus* leaves (Figure 2) have been applied in sausages and meat products, used to prevent fungal contamination during fig drying and to control nematodes in stored products [12,13]. These examples underscore the potential of *Pistacia* leaves as natural preservatives, offering a sustainable alternative to synthetic additives.

Wild *Pistacia* species have a long history of use in traditional medicine for treating a range of ailments—from asthma and diabetes to gastrointestinal disorders—because of their high bioactive content [3,7,14]. This extensive use suggests a favorable safety profile, as recent studies indicate that extracts from *Pistacia* leaves exhibit low cytotoxicity towards normal cells, making them suitable for food applications. For example, these extracts have demonstrated protective effects against oxidative stress without adversely affecting cellular health at recommended dosages [15,16,17,18,19]. Toxicological studies reinforce this safety, revealing minimal toxicity even at higher concentrations. Notably, *P. terebinthus* and *P. lentiscus* leaves, widely used in Algerian folk medicine, were analyzed using instrumental neutron activation analysis. The analysis confirmed that the mass fractions of sixteen trace elements are within the World Health Organization’s tolerance limits, providing a scientific basis for their optimal usage and enhancing the database of medicinal herbs [20].

Harnessing cultivated *Pistacia* species provides a valuable method for utilizing their by-products and enhancing the sustainability of pistachio farming. Incorporating wild species, with their unique properties and potential uses, further helps optimize resource use and supports a circular agricultural economy [21].

This review explores the potential of *Pistacia* leaves as natural food preservatives, highlighting their functional properties and health benefits. It advocates for their integration into food systems as a sustainable method to enhance food quality and safety. Additionally, this review aims to inspire further research on *Pistacia* leaves to advance their utilization.

This review will discuss the following themes:

1. Taxonomy of the plants within the *Pistacia* genus.
2. Traditional and ethnopharmacological uses of *Pistacia* leaves.
3. Antioxidant activity of *Pistacia* leaf extracts.
4. Antimicrobial activity of *Pistacia* leaf extracts.
5. Bioactive compounds in the *Pistacia* leaf extracts.
6. Importance of the extraction methods.
7. Potential food applications of *Pistacia* leaf extracts.

## 2. Methodology

A comprehensive search was performed across several scientific databases, including PubMed, Scopus, Web of Science, and Google Scholar. The search utilized a combination of keywords such as “*Pistacia* leaves”, “*Pistacia vera*”, “wild *Pistacia* species”, “*Pistacia lentiscus*”, “*Pistacia atlantica*”, “*Pistacia terebinthus*”, “*Pistacia palaestina*”, “*Pistacia chinensis*”, *Pistacia integerrima*”, “*Pistacia khinjuk*”, “*Pistacia weinmannifolia*”, antioxidant activity”, “antimicrobial activity”, “food preservation”, and “bioactive compounds”.

The inclusion criteria were as follows:

- Articles published in English.
- Studies focusing on *Pistacia* species leaves with clear analyses of antioxidant and/or antimicrobial activities.
- Papers employing relevant extraction methods of the leaves, including ultrasound-assisted extraction (UAE), microwave-assisted extraction (MAE), supercritical carbon dioxide extraction (scCO2), or conventional methods.
- Studies that were published primarily between 2017 and the cutoff date of June 2024.
- Studies that evaluated the food preservation potential of plant extracts.

Exclusion criteria included:

- Articles not available in full text.
- Studies that focus solely on the nuts or other parts of the tree.
- Papers not directly related to the antioxidant and antimicrobial properties of *Pistacia* species leaves or the other themes discussed.

In total, 71 literature sources were included in this article. The species of *Pistacia* covered in this review include *P. vera*, *P. atlantica*, *P. terebinthus*, *P. palaestina*, *P. integerrima*, *P. chinensis*, *P. khinjuk*, and *P. lentiscus*, originating from countries such as Iran, Pakistan, Saudi Arabia, Syria, Turkey, Greece, Croatia, Italy, Libya, Algeria, Tunisia, and Morocco.

This review was conducted between July and September 2024, and all studies published up until the end of June 2024 were considered in the analysis.

## 3. Results

### 3.1. Taxonomy of the Species within the Pistacia Genus

Table 1 provides the taxonomic classification and geographical distribution of the *Pistacia* species discussed in this study [22]. The taxonomy offers a structured framework for understanding the evolutionary relationships among the species, while distributional data underscore their ecological adaptability and biogeographical spread.

The inclusion of both taxonomic ranks and distribution information highlights the global occurrence of the *Pistacia* genus, from Mediterranean and Middle Eastern regions to parts of Asia and North Africa. This geographic variability is significant in understanding the species’ adaptability to different climates and ecological conditions, which may have implications for conservation, horticultural practices, or their use in traditional medicine.

### 3.2. Traditional and Ethnopharmacological Uses of Pistacia Leaves

The leaves of *Pistacia* species are highly valued for their diverse traditional and ethnopharmacological applications across cultures, reflecting their therapeutic potential and safety for consumption. Their extensive use in food, medicine, and livestock fodder supports their integration into various healing practices and culinary traditions, primarily as raw material, infusions, decoctions, and topical preparations [13].

*Pistacia vera* leaves, for example, are traditionally used for their analgesic, astringent, antitussive, diuretic, and expectorant properties, highlighting their role in managing pain, coughs, and fluid retention [23]. In Turkish cuisine, *P. atlantica* leaves are consumed as a vegetable and are also recognized for their antidiabetic effects, as well as their use in treating stomach aches and eye infections [24].

*Pistacia terebinthus* leaves are valued for their astringent and antidiarrheal properties, acting as diuretics and antihypertensives, and are used to treat jaundice, stomach aches, mycosis, and diabetes. Historically, these leaves have also served as a ritual food in Iran [24]. Meanwhile, old leaves of *P. palaestina* have been used for the treatment of mental disorders, blood system disorders, sunstroke, injuries, and wounds [13]. The leaves of *P. integerrima* have long been utilized in traditional remedies to treat cough, asthma, fever, jaundice, diarrhea, and even snake bites [25]. Decoctions of *P. khinjuk* have been used for treating bone pain and mental disorders [13].

In South Asia, *P. chinensis* is recognized for its role in managing asthma, diarrhea, diabetes, liver diseases, fever, pain, and inflammation [26]. Meanwhile, *P. lentiscus* leaves have been widely employed in Mediterranean medicine for a broad range of conditions, from digestive disorders and bronchitis to osteoarthritis, allergies, and wound healing. Their antipyretic, astringent, and antimicrobial properties further enhance their therapeutic potential, with applications in treating eczema, renal stones, and oral health issues. In culinary contexts, *P. lentiscus* leaves are used to soothe heartburn, as appetizers, and to promote overall digestive health [7,24].

Overall, the diverse traditional and ethnopharmacological applications of *Pistacia* leaves underscore their significance in various cultures’ healing systems. Their widespread medicinal and culinary use reflects a strong safety profile, further supported by their therapeutic efficacy in addressing numerous ailments.

### 3.3. Potential Use of Pistacia Leaves as Food Preservatives

Plant extracts with potential as natural preservatives can prevent oxidation, which helps reduce rancidity and spoilage in food products, and/or inhibit microbial growth, which helps prevent the growth of harmful bacteria, yeast, and molds. This dual action extends food shelf life and enhances safety [27].

#### 3.3.1. Antioxidant Activity

The antioxidant activity of *Pistacia* leaf extracts has been extensively evaluated in vitro using colorimetric assays such as 2,2-diphenyl-1-picrylhydrazyl (DPPH), 2,2’-azino-bis(3-ethylbenzothiazoline-6-sulfonic acid) (ABTS), ferric reducing antioxidant power (FRAP), cupric ion reducing antioxidant capacity (CUPRAC), and oxygen radical absorbance capacity (ORAC). Additionally, the potency of these extracts has been assessed against hydrogen peroxide (H_2_O_2_), hydroxyl radicals (•OH), superoxide anion radicals (O_2_•^−^), carotene bleaching (CB), metal chelating capacity (MCC), and lipid peroxidation (LP). The results are summarized in Table 2.

##### *Pistacia* *vera*

Extracts from male and female leaves derived by UAE with 50% ethanol (EtOH) demonstrated high antioxidant properties: over 600 mg Trolox equivalents (TE)/g dry weight (DW) in the DPPH assay, more than 1300 mg TE/g DW in the ABTS assay, and over 500 mg TE/g DW in the FRAP assay. Male leaves were primarily composed of chlorophylls, limonene, and palmitic acid, while female leaves were rich in syringaldehyde and limonene [8,9]. In contrast, the EtOH extracts obtained via MAE exhibited roughly half the antioxidant activity compared with UAE, underscoring the influence of the extraction method on bioactivity. The main components identified were myricetin galloyl hexoside in the male leaves and apigenin and quercetin-hexoside in the female leaves [9]. An 80% EtOH extract obtained through conventional extraction demonstrated superior radical-scavenging activity compared with Trolox, with half maximal inhibitory concentration (IC_50_) values of 44.08 µg/mL for DPPH and 139.84 µg/mL for ABTS. Major components included phloroglucinol, gallic acid, and vanillic acid. Additionally, the extract exhibited potent in vitro antidiabetic, anti-hypercholesterolemic, and anti-obesity effects, reflecting the recognized role of oxidative stress in these conditions [28].

##### *Pistacia* *atlantica*

The ethyl acetate (EtOAc) fraction of the methanol (MeOH) extract exhibited antioxidant activity that was either stronger or comparable to butylated hydroxyanisole (BHA) and butylated hydroxytoluene (BHT), with IC_50_ values of 0.99 µg/mL for DPPH and 1.54 µg/mL for ABTS. It also showed half maximal absorbance (A_0.5_) values of 4.98 µg/mL in the CUPRAC assay and 0.85 µg/mL in the FRAP assay, attributable to high concentrations of gallic and cichoric acids. The extract also showed significant antiproliferative activity against the HeLa cell line [29]. Ethyl acetate and MeOH extracts showed potent activity against DPPH radical with IC_50_ values of 1.82 μg/mL and 8.41 μg/mL, respectively. Six phenolic compounds were identified, likely contributing to the radical scavenging activity. These include three polyphenolic acids—gallic, ellagic, and 3,3′-dimethoxyellagic acids—as well as three gallotannins—1,3-di-*O*-galloyl-β-D-^4^C_1_-glucopyranose, 1,2,3,4,6-penta-*O*-galloyl-β-D-^4^C_1_-glucopyranose, and nilocitin [30].

##### *Pistacia* *terebinthus*

The EtOAc, MeOH, and water (H_2_O) extracts displayed total phenolic contents ranging from 65.51 to 210.54 mg GAE/g. These extracts demonstrated significant antioxidant capacity with IC_50_ values of 0.54, 3.29, and 2.66 mmol TE/g (ABTS); 0.29, 2.06, and 1.88 mmol TE/g (DPPH); 0.58, 2.67, and 1.82 mmol TE/g (CUPRAC); and 0.29, 1.62, and 1.25 mmol TE/g (FRAP). Additionally, the extracts exhibited metal chelating activity with values of 64.43, 65.65, and 13.13 µmol ethylenediaminetetraacetic acid (EDTA)/g. The common compounds identified in all three extracts include quinic acid, gallic acid, rutin, and luteolin. Phylloquinone, pheophytin B, and pheophytin A were found exclusively in the EtOAc extract, while procyanidin B, catechin, and epigallocatechin-3-*O*-gallate were present only in the MeOH and H_2_O extracts. The extracts exhibited strong inhibitory activity against acetylcholinesterase, butyrylcholinesterase, α-amylase, α-glucosidase, and tyrosinase, indicating potential therapeutic applications for global health issues such as skin hyperpigmentation, Alzheimer’s disease, and diabetes mellitus [31].

##### *Pistacia* *palaestina*

The antioxidant activities of the 96% EtOH extract, rich in gallotannins, were reported as 23.5 mmol Fe^2+^/g (FRAP), 4562 µmol TE/g (CUPRAC), 344 µmol/g (DPPH), and 53.1 µmol/g (ABTS). The main constituents identified were quinic and gallic acids, as well as gallotannins [32]. Notably, young leaves of *P. palaestina* contained higher levels of phenolic compounds than mature leaves. Among the solvents tested, 40% acetone was the most effective for extracting phenolic compounds, achieving a superior DPPH antioxidant capacity of 99.69% [33].

##### *Pistacia* *chinensis*

The EtOH extract exhibited scavenging activity against DPPH and hydrogen peroxide with IC_50_ values of 39.4 and 21.9 µg/mL, respectively, similar to ascorbic acid. However, it demonstrated lower efficacy in the ABTS assay (IC_50_ = 205.0 µg/mL), CB test (IC_50_ = 202.1 µg/mL), and in scavenging O_2_•^−^ (IC_50_ = 120.0 µg/mL), •OH (IC_50_ = 234.0 µg/mL), and LP (IC_50_ = 198.0 µg/mL) compared with the reference compound [34]. The EtOAc and butanol (BuOH) extracts of edible tender leaves exhibited higher DPPH scavenging activities than BHA and comparable efficacy to ascorbic acid, suggesting they could be valuable new sources of natural antioxidants [35].

##### *Pistacia* *lentiscus*

The aerial parts of *P. lentiscus* have been extensively examined. Across various studies, extracts obtained using different methods have demonstrated potent antioxidant activity. An acetone extract containing key components such as gallic acid, quercetin, and catechin demonstrated significant DPPH radical inhibition with IC_50_ values of 0.0015 mg/mL for the extract, 0.0068 mg/mL for gallic acid, 0.0070 mg/mL for quercetin, and 0.0148 mg/mL for catechin. The extract activity, comparable to ascorbic acid (IC_50_ = 0.0018 mg/mL), suggests potential synergistic interactions among the constituents [36]. The EtOH extract exhibited significant DPPH activity with an IC_50_ of 5.44 µg/mL, while the MeOH extract demonstrated high FRAP activity with an IC_50_ of 309.6 mg/g ascorbic acid equivalents (AAE) [37]. The decoction extract demonstrated 58.68% DPPH scavenging capacity and IC_50_ values of 99.72 mg/mL in the ABTS assay and 19.99 mg/mL in the FRAP assay [38]. Soxhlet extraction with 70% EtOH yielded 50.42% phenolic content, with major components including 3,5-di-*O*-galloylquinic acid and gallic acid. The extract exhibited superior DPPH scavenging activity, with a half maximal effective concentration (EC_50_) of 12.588 µg/mL, outperforming ascorbic acid. Additionally, it demonstrated promising in vitro antidiabetic and anticoagulant properties [39]. Ethanolic and H_2_O extracts demonstrated significant antioxidant activities comparable to BHT and ascorbic acid. The DPPH scavenging IC_50_ values were 10.84 µg/mL for the EtOH extract and 15.85 µg/mL for the H_2_O extract, while the FRAP values were 5.32 µg/mL and 5.37 µg/mL, respectively. Both extracts also exhibited notable anti-inflammatory effects, including significant inhibition of nitric oxide production and key inflammatory enzymes such as cyclooxygenase and myeloperoxidase [40]. Microwave-assisted extraction with 70% EtOH yielded 108.14 mg gallic acid equivalents (GAE)/g phenolic content and an ORAC value of 538.41 µmol TE/g. Significant compounds included myricetin rhamnoside, myricetin glucuronide, digalloylquinic acid, and myricetin rutinoside [41].

A lactic acid extract, rich in phenolic compounds, exhibited antioxidant activity by scavenging DPPH and ABTS radicals with IC_50_ values of 186.39 and 176.72 mmol TE/100 g DW, respectively. It was also effective against •OH with an IC_50_ of 171.11 mg AAE/g DW and demonstrated a FRAP value of 278.41 mg AAE/g DW. Additionally, the extract showed a chelating power of 118.83 mg EDTAE/g DW [42]. Chelating agents bind to and remove toxic metals, thereby reducing harmful effects and enhancing the bioavailability of essential metals, which is beneficial for both plant health and human nutrition [43,44].

Supercritical carbon dioxide (scCO2) extract has antioxidant and protective effects against hydrogen peroxide-induced oxidative stress. With its safe profile and potent antioxidant and vascular protective properties, the extract shows potential for use in developing effective vascular health supplements and treatments. The main components identified were germacrene D, δ-cadinene, and α-pinene [17].

#### 3.3.2. Antimicrobial Activity

*Pistacia* leaf extracts have been tested against a broad spectrum of bacteria and fungi, including foodborne and spoilage pathogens. The tested microorganisms include Gram-positive bacteria (*Staphylococcus aureus*, *Enterococcus faecalis*, *Bacillus subtilis*, *Bacillus cereus*, *Streptococcus mutans*, *Streptococcus mitis*, *Listeria monocytogenes*, *Listeria innocua*, and *Micrococcus luteus*), Gram-negative bacteria (*Escherichia coli*, *Pseudomonas aeruginosa*, *Citrobacter freundii*, *Klebsiella pneumoniae*, *Shigella flexneri*, *Salmonella typhi*, *Salmonella paratyphi*, *Salmonella enterica*, *Acinetobacter baumannii*, *Proteus mirabilis,* and *Vibrio cholerae*), fungi (*Candida albicans*, *Candida kefyr*, *Candida tropicalis*, and *Saccharomyces cerevisiae*), and molds (*Mucor circinelloides*, *Rhizopus oryzae*, *Fusarium oxysporum* f. sp. *albedinis*, and *Aspergillus niger*). The results are summarized in Table 3.

##### *Pistacia* *vera*

The extracts from male and female leaves, obtained using UAE in 50% MeOH, exhibited substantial antimicrobial activity against various microorganisms, including *Streptococcus* spp., *S*. *aureus*, *E*. *faecalis*, *C. freundii*, *E*. *coli*, *K*. *pneumoniae*, and *C*. *albicans*. The female leaf extract notably inhibited microbial growth in fortified bread, highlighting its potential as a natural food preservative. Additionally, leaf extracts from both genders, obtained via MAE in 70% EtOH, showed minimum inhibitory concentrations (MICs) between 0.04 and 0.3 mg/mL, with strong efficacy against *K. pneumoniae* [11].

##### *Pistacia* *integerrima*

The crude MeOH extract and its sub-fractions were evaluated for antimicrobial activity against *P*. *aeruginosa*, *E. coli*, *S*. *flexneri*, *S*. *typhi*, *S. aureus*, and *B*. *subtilis* using the agar well diffusion method. The crude extract, along with EtOAc and *n*-BuOH fractions, exhibited significant antibacterial activity. The EtOAc fraction contained bioactive compounds such as methyl benzoate, 1,5-dihydroxy-3-methylanthraquinone, gallic acid, betulin, 6,7,8-trimethoxycoumarin, rosmarinic acid, methyl rosmarinate, and olean-12(13)-en-3β-hexadecanoate [55]. Another study found that the MeOH extract was more active than the EtOH and H_2_O extracts against *B. subtilis*, *E. coli*, *P. aeruginosa*, and *S. aureus* at a concentration of 200 µL [45]. The EtOH extract exhibited antibacterial activity, with MIC values of 166.6 mg/mL against *S. aureus*, coagulase-negative *Staphylococcus*, and *E. coli*, and 133.3 mg/mL against *P. aeruginosa*, *S. typhi*, and *K. pneumoniae* [46].

##### *Pistacia* *atlantica*

Phytochemicals are potent antimicrobial agents, particularly because of their ability to inhibit quorum sensing (QS)—a bacterial communication mechanism that regulates virulence gene expression. Disrupting QS can reduce bacterial pathogenicity, offering a promising alternative to traditional antibiotics [56]. For example, the MeOH leaf extract of *Pistacia atlantica* significantly inhibited biofilm formation and pigment production in the multidrug-resistant *P. aeruginosa* strain PAO1, with minimum biofilm inhibitory concentration (MBIC) of 0.06 mg/mL and MIC of 0.50 mg/mL, respectively. Flavonoids such as myricetin-3-*O*-rutinoside, rutin, isoquercitrin, and kaempferol-3-*O*-rutinoside achieved over 90% inhibition of biofilm formation and reduced pyocyanin production by 40-70% [47]. Additionally, the EO exhibited inhibitory activity against *E. coli*, *K. pneumoniae*, and *A*. *baumannii*, with MIC values ranging from 0.022 to 0.044 mg/mL and minimum bactericidal concentration (MBC) values of 0.044 mg/mL. This activity is primarily attributable to components such as terpinen-4-ol and spathulenol [48].

##### *Pistacia* *khinjuk*

Biogenic silver (Ag) nanoparticles synthesized from *P. khinjuk* leaf extract in deionized water demonstrated potent antibacterial and antifungal activities, with MIC values of 0.58 µg/mL against *E. coli*, *K. pneumoniae*, and *S. aureus*; 1.17 µg/mL against *S. mutans* and *E. faecalis*; 2.34 µg/mL against *S. mitis*; 0.15 µg/mL against *C. albicans* [49]. These biosynthesized nanoparticles have potential applications in the food industry, particularly as chelating agents in food packaging to enhance product stability and quality by binding metal ions that could otherwise support spoilage microorganisms [57].

##### *Pistacia* *chinensis*

The 80% MeOH extract demonstrated antibacterial activity against *B. cereus* and *S. paratyphi* with inhibition zones of 25 mm in a disc-diffusion assay. This activity is attributed to its principal components: β-sitosterol, lupeol, quercetin, myricetin, quercetin 3-*O*-α-rhamnoside, quercetin 3-*O*-β-glucoside, myricetin 3-*O*-α-rhamnoside, and myricetin 3-*O*-β-glucuronide [50]. The extract shows potential for use in the preservation of various foods, particularly in meat and dairy products, by inhibiting microbial growth and extending shelf life.

##### *Pistacia* *lentiscus*

The EO exhibited significant inhibitory effects against *P*. *mirabilis* and *L*. *monocytogenes*, comparable to chloramphenicol, with MIC and MBC values of 0.25% (*v*/*v*) against *P. aeruginosa* and *P. mirabilis*. This potency is largely attributed to its main components, β-myrcene, and α-pinene, which can damage the cytoplasmic membrane of *P. aeruginosa*, influencing its growth and viability [18,51]. The EO of *Pistacia lentiscus*, rich in D-limonene, 3-carene, and β-myrcene, has shown significant potential as a natural food preservative. It was evaluated alongside the EOs of lemon eucalyptus (containing citronellal, β-citronellol, and 1,8-cineole) and sweet orange (rich in D-limonene). Interestingly, several formulations were optimized to leverage the synergistic effects of these essential oils, enhancing antimicrobial activity against foodborne pathogens such as *C. albicans*, *S. aureus*, *E. coli*, *S. enterica*, and *B. cereus* [52].

Leaf extracts obtained via UAE with EtOAc, MeOH, and H_2_O exhibited antifungal activity. Notably, the main components, myricitrin and 2-hydroxy-1,8-cineole β-D-glucopyranoside, showed selective antifungal effects against *M*. *circinelloides* and *R*. *oryzae* [58]. Acetone macerates significantly inhibited the growth of *S. aureus* and *P. aeruginosa* in a dose-dependent manner, as demonstrated by the agar well diffusion method. This antibacterial activity was primarily attributable to the main constituents, pyrogallol, and quercetin [59]. Aqueous macerates inhibited both *S. aureus* and *L. innocua*, with MIC and MBC values of 2.23 and 4.46 mg/mL, respectively. The extract was incorporated into an adhesive, resulting in a novel active multilayer packaging [38]. The ethyl acetate and acetone extracts demonstrated significant antimicrobial activity against various bacterial strains. Specifically, the extracts showed relative inhibition (RI) values of 70% and 75% against *M*. *luteus*, 60% for both extracts against *L. innocua*, and 60% and 50% against *E. coli*, respectively. These results indicate the effectiveness of both extracts in inhibiting bacterial growth. Additionally, the EtOAc and H_2_O extracts demonstrated potential in preventing microbial spoilage of dates by inhibiting the growth of *F*. *oxysporum* f. sp. *albedinis* [37]. Acetone and MeOH extracts of *P. lentiscus* also exhibited dose-dependent antimicrobial activity against several microorganisms, including *S. aureus*, *P. aeruginosa*, and *L. monocytogenes*, with MIC values ranging from 1.25 to 2.5 µg/mL and MBCs of 5 µg/mL [53]. The flavonoid extracts showed strong activity against *C. albicans*, *V*. *cholerae*, *S. aureus*, and methicillin-resistant *S. aureus* (MRSA), with MIC and either minimum fungicidal concentration (MFC) or MBC values of 0.1 (5.0), 0.3 (0.5), 2.0 (3.0), and 0.5 (1.0) mg/mL, respectively. This activity is attributable to their ability to disrupt nucleic acid synthesis and cytoplasmic membrane function [54,60]. Additionally, an H_2_O extract rich in galloyl quinic acids demonstrated effectiveness against *K. pneumoniae* (MIC = 0.6 mg/mL, MBC = 1.2 µg/mL) and *Salmonella* spp. (MIC = 1.2 mg/mL, MBC = 2.5 mg/mL). This extract also exhibited superior activity against foodborne yeasts such as *C. albicans*, *C. kefyr*, *C. tropicalis*, and *S*. *cerevisiae*, as well as the mold *A*. *niger*, compared with terbinafine [39]. This indicates its potential application in preserving a variety of food products, including dairy items, baked goods, fruits, vegetables, and foods prone to *Salmonella* contamination, such as poultry, eggs, and raw meat.

## 4. Discussion

This review highlights the significant potential of *Pistacia* leaves as natural food preservatives, focusing on their antioxidant and antimicrobial properties. A detailed comparative analysis of *Pistacia vera* and wild *Pistacia* species is impeded by inconsistencies in the presentation of results. Nonetheless, notable differences in bioactivity are observed, suggesting that species-specific traits influence their preservative effectiveness.

### 4.1. Antioxidant Activity

*Pistacia vera* leaves exhibit robust antioxidant properties, especially when extracted via UAE and MAE. These techniques enhance the yield of key antioxidants, including flavonoids and phenolic acids. Extracts from *P. vera* demonstrate high antioxidant capacity in assays such as DPPH, ABTS, and FRAP, suggesting their potential to delay lipid oxidation and prolong the shelf life of lipid-containing foods [8,9].

**Figure 3:**
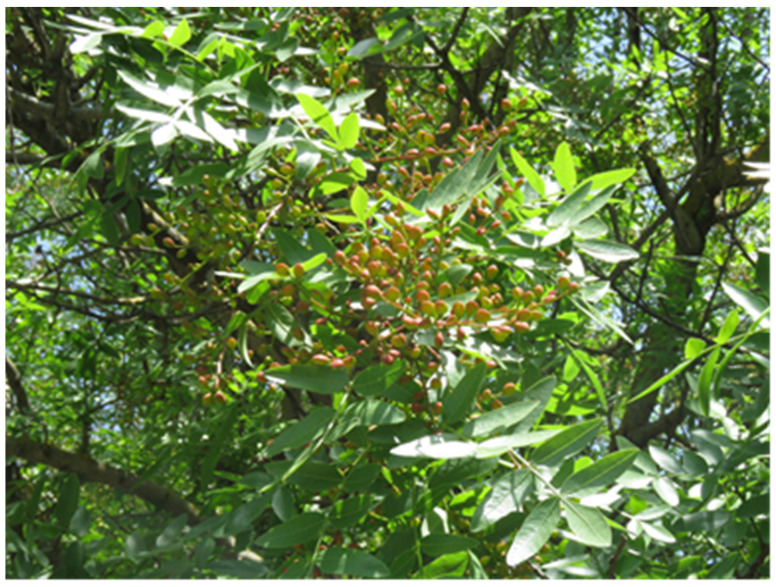
Leaves and fruits of *Pistacia atlantica*.

Wild *Pistacia* species often show antioxidant activities that can exceed those of *P. vera*. For instance, *P. atlantica*, *P. chinensis*, and *P. lentiscus* leaf extracts offer antioxidant properties comparable to or even better than ascorbic acid and synthetic preservatives such as BHA and BHT [29,35,40]. The high antioxidant activity of certain *Pistacia* extracts can be attributed to synergistic interactions among their various phenolic constituents. For example, the 80% acetone extract of *P. lentiscus* demonstrated higher antioxidant activity than any of its individual components (gallic acid, quercetin, and catechin), suggesting that the combined effect of these compounds in the extract enhances its overall bioactivity [36]. Similarly, *P. atlantica* leaves (Figure 3) exhibit substantial antioxidant effects because of their high concentrations of phenolic acids, flavonoids, and tannins [29,30].

Oxidative stress is linked to numerous conditions, including inflammation, diabetes, cancer, and neurodegenerative diseases [61]. Due to their high antioxidant activities, some of these extracts have been studied for other health benefits, revealing promising results that suggest their potential as ingredients in functional foods.

### 4.2. Antimicrobial Activity

Beyond their antioxidant benefits, *P. vera* leaves also display notable antimicrobial activity. Extracts and EOs from *P. vera* effectively inhibit various foodborne pathogens, demonstrating substantial control over bacterial growth and spoilage microorganisms [8,9].

Wild species such as *P. lentiscus* and *P. atlantica* are particularly distinguished by their potent antimicrobial properties. Essential oil from *P. lentiscus* effectively disrupts the cytoplasmic membranes of pathogens such as *P. aeruginosa* and *P. mirabilis* [18]. Its flavonoid extracts also interfere with nucleic acid synthesis and membrane function in various microorganisms, including resistant strains [54]. Additionally, *P. lentiscus* H_2_O extract shows impressive activity against foodborne yeasts, emphasizing its potential for food preservation [39]. *Pistacia atlantica* is valuable for addressing bacterial resistance, significantly inhibiting quorum sensing in *P. aeruginosa* [47]. Tannins in both *P. lentiscus* and *P. atlantica* contribute to antimicrobial effects. These phenolic compounds act through mechanisms such as iron chelation, cell wall synthesis inhibition, membrane disruption, and interference with fatty acid biosynthesis, as well as by attenuating gene expression related to virulence factors for biofilms, enzymes, adhesins, motility, and toxins [62].

### 4.3. Bioactive Compounds in Pistacia Leaves

**Figure 4:**
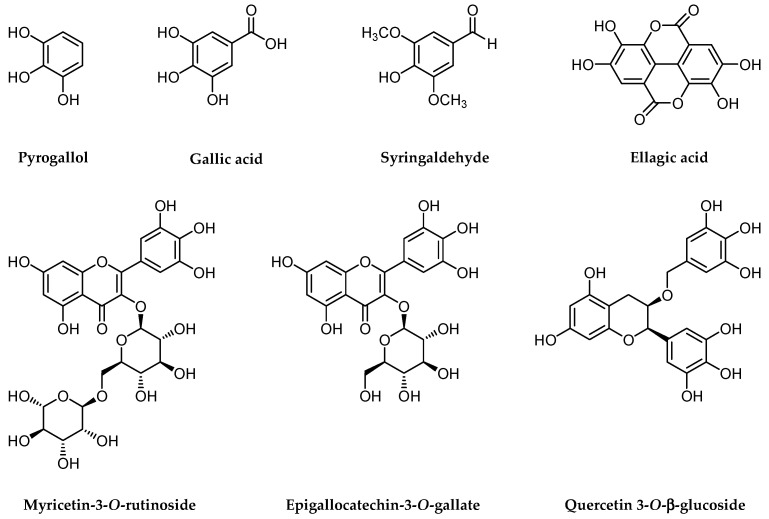
Phenolic Compounds in Pistacia Leaves with Antioxidant and Antimicrobial Properties.

Leaves of *Pistacia* species are rich in antioxidant and antimicrobial components, including EOs, triterpenoids, and a diverse array of phenolic compounds. These include flavonoids, tannins, and phenolic acids [3,14]. Collectively, these compounds regulate physiological and developmental processes while providing protection against abiotic factors (e.g., radiation), pathogens, and herbivores. The chemical composition of leaves, in terms of both quantity and quality, can vary markedly among individuals and populations. Factors such as genotype, geographic location, environmental conditions (e.g., soil type, climate, altitude), and the plant’s developmental stage contribute to this intraspecific chemical variation [7,63,64,65,66,67]. Figure 4 illustrates representative phenolic compounds found in *Pistacia* leaves, which demonstrate potent antioxidant and/or antimicrobial properties.

### 4.4. Extraction Methods

Extraction methods are pivotal in influencing the bioactive potential of *Pistacia* leaf extracts and their food applications. Advanced techniques such as UAE and MAE yield higher concentrations of bioactive compounds compared with traditional methods such as Soxhlet extraction and maceration [8,9,11,18,38,58,67]. For instance, UAE, with 50% EtOH, has been shown to produce extracts with superior antioxidant activity compared with conventional methods, highlighting the advantages of optimizing extraction parameters [9]. Furthermore, scCO2 has been scaled up to obtain essential oil from *P. lentiscus* leaves, demonstrating a safe profile and antioxidant capacity. This method could also be successfully applied to other species within the genus [17].

The choice of solvent and extraction conditions also plays a crucial role in determining the extract composition, with solvents such as EtOH, acetone, and H_2_O demonstrating varying effectiveness in scavenging radicals and inhibiting microbial growth. This variability underscores the need for careful selection of solvents and extraction methods tailored to the intended application, whether it be for enhancing food quality or extending shelf life. Notably, *Pistacia* leaf extracts often serve dual roles as antioxidants and antimicrobial agents, making them particularly valuable for improving food quality and prolonging shelf life. Table 4 summarizes the potential food applications of *Pistacia* leaf extracts, considering the extraction methods used and the key bioactive components.

Despite these advancements, standardized extracts of *Pistacia* leaves are not yet commercially available. Developing such formulations is crucial for ensuring consistent quality and potency, which would facilitate reliable evaluations and promote broader use in functional foods and supplements [68,69].

### 4.5. Potential Food Applications of Pistacia Leaf Extracts

*Pistacia* leaf extracts, similar to many polyphenol-rich plant-based extracts with potent antioxidant and antimicrobial properties, hold significant promise as natural food preservatives [70,71]. These extracts can be directly incorporated into various food matrices, with their efficacy largely dependent on the chemical composition of the leaves and the bioactivity-guided extraction methods used. It is also essential that the extracts are generally recognized as safe (GRAS) and that further research is conducted using in vivo models to evaluate their effectiveness and safety [71].

However, there are limitations to the use of *Pistacia* leaves. Their efficacy may diminish during high-temperature food processing methods, such as cooking or pasteurization, as some bioactive compounds may degrade under heat. Additionally, the preservative activity of *Pistacia* extracts may decline over extended storage periods due to the breakdown of active compounds, particularly in non-refrigerated environments [71]. Further research is needed to optimize formulations for heat-stable applications and to evaluate their long-term preservative effectiveness across various food products and packaging systems.

In addition to direct incorporation into foods, *Pistacia* leaf extracts can also be used as ingredients in food packaging materials. For example, macerated *P. lentiscus* leaves have been successfully integrated into packaging systems. In these studies, extracts at concentrations of 1% and 2% were prepared in a water-based adhesive, maintaining the adhesive’s homogeneity and adhesion properties. These extracts were then applied in a multilayer active packaging system consisting of low-density polyethylene (LDPE), which directly contacts the food, and polyethylene terephthalate (PET) as the outer protective layer. This approach not only enhances the preservative qualities of the packaging but also complies with European Union regulations for food safety, as the adhesive used is approved for food contact [38].

Moreover, *P. khinjuk* leaf extract has been used as a capping and reducing agent in the green synthesis of Ag nanoparticles. These biogenic nanoparticles have shown strong antibacterial activity against both Gram-negative and Gram-positive microorganisms, along with significant antioxidant properties [49]. The synthesis of these nanoparticles highlights the potential of *Pistacia* leaf extracts, not only as natural preservatives but also as components in active packaging that can enhance food safety and extend shelf life.

## 5. Conclusions

This review underscores the significant potential of *Pistacia* leaves as natural food preservatives, emphasizing their antioxidant and antimicrobial properties. The comparative analysis between *Pistacia vera* and wild *Pistacia* species reveals notable species-specific differences in bioactivity, which influence their effectiveness in food preservation. The antioxidant activity of *P*. *vera* leaves, particularly when extracted using advanced techniques, demonstrates a high capacity to delay lipid oxidation, thus extending the shelf life of lipid-containing foods.

Wild *Pistacia* species, such as *P*. *lentiscus* and *P*. *atlantica*, exhibit even greater antioxidant and antimicrobial activities, making them valuable candidates for further study. Their rich array of bioactive compounds, including EOs, flavonoids, and phenolic acids, contributes to protective effects against oxidative stress and foodborne pathogens.

However, the effectiveness of these extracts is significantly influenced by extraction methods and solvent choice, highlighting the need for optimized techniques to maximize bioactive yields. While there is potential for commercial applications of standardized extracts, rigorous safety evaluations and adherence to regulatory guidelines are essential to ensure consumer safety.

Future research should focus on exploring a broader range of *Pistacia* species, refining extraction techniques, assessing stability, and evaluating the health benefits and functional properties of these extracts. By advancing our understanding of their preservative capabilities, *Pistacia* leaf extracts may play a pivotal role in enhancing food quality and safety in the functional foods market.

## Figures and Tables

**Table 1:** Taxonomy and Geographical Distribution of *Pistacia* Species in This Study.

Genus Sections	Species in the Sections	Geographical Distribution
Terebinthus	*P. atlantica* Desf.	From North Africa to the Iranian Plateau
	*P. chinensis* Bunge	Central and Western China
	*P. integerrima* J.L. Stew. ex. Brandis	Asia
	*P. khinjuk* Stocks	Egypt, Western Asia, and parts of the Himalayas,
	*P. terebinthus* L.	Morocco, and Portugal to Greece, Western and Southeast Turkey, and the Levant region
	*P. palaestina* (considered a sibling of *P. terebinthus* L.)	East Mediterranean (Israel, Syria, Lebanon)
	*P. vera L.*	Central Asia and the Middle East
Lentiscus	*P. lentiscus* L.	Mediterranean Basin

**Table 2:** Antioxidant Activity of Various *Pistacia* Species Leaf Extracts as Determined by Different Assays.

*Pistacia* Species	Antioxidant Assay	IC_50_	Unit	References
*P. vera*	DPPH ABTS	>600 >1300	mg TE/g DW mg TE/g DW	[8,9]
	FRAP	>500	mg TE/g DW	
	DPPH	44.08	µg/mL	[28]
	ABTS	139.84	µg/mL	
*P. atlantica*	DPPH	0.99	µg/mL	[29]
	ABTS	1.54	µg/mL	
	CUPRAC	4.98 *	µg/mL	
	FRAP	0.85 *	µg/mL	
	DPPH	1.82; 8.41	μg/mL	[30]
*P. terebinthus*	DPPH	0.29; 2.06; 1.88	mmol TE/g	[31]
	ABTS	0.54; 3.29; 2.66	mmol TE/g	
	CUPRAC	0.58; 2.67; 1.82	mmol TE/g	
	FRAP	0.29; 1.62; 1.25	mmol TE/g	
	MCC	64.43; 65.65; 13.13	µmol EDTA/g	
*P. palaestina*	FRAP	23.5	mmol Fe^2+^/g	[32,33]
	CUPRAC	4562	µmol TE/g	
	DPPH	344; 99.69	µmol/g; %	
	ABTS	53.1	µmol/g	
*P. chinensis*	DPPH	39.4; 93.16; 93.71	μg/mL; %; %	[34,35]
	H_2_O_2_	21.9	µg/mL	
	ABTS	205.0	µg/mL	
	CB	202.1	µg/mL	
	O_2_•^−^	120.0	µg/mL	
	•OH	234.0	µg/mL	
	LP	198.0	µg/mL	
*P. lentiscus*	DPPH	1.5	µg/mL	[36]
	DPPH	5.44	µg/mL	[37]
	FRAP	309.6	mg/g AAE	
	DPPH	58.68	%	[38]
	ABTS	99.72	mg/mL	
	FRAP	19.99	mg/mL	
	DPPH	12.59 **	µg/mL	[39]
	DPPH	10.84; 15.85	µg/mL	[40]
	FRAP	5.32; 5.37	µg/mL	
	ORAC	538.41	µmol TE/g	[41]
	DPPH	186.39	mmol TE/100 g DW	[42]
	ABTS	176.72	mmol TE/100 g DW	
	•OH	171.11	mg AAE/g DW	
	FRAP	278.41	mg AAE/g DW	
	MCC	118.83	mg EDTAE/g DW	

* A_0.5_—half maximal absorbance; ** EC_50_—half maximal effective concentration.

**Table 3:** Antimicrobial Activity of *Pistacia* Species Leaf Extracts Against Various Microorganisms.

*Pistacia* Species	Tested Microorganisms	MIC	Reference
*P. vera*	*S. aureus* *E. faecalis, B Streptococcus* *C. freundii* *E. coli* *K. pneumoniae* *C. albicans*	26 mm (10 µL/disc) ^a^ 19 mm (10 µL/disc) ^a^ 24 mm (10 µL/disc) ^a^ 22 mm (10 µL/disc) ^a^ 20 mm (10 µL/disc) ^a^ 25 mm (10 µL/disc) ^a^	[11]
*P. integerrima*	*S. aureus* *B. subtilis* *E. coli* *P. aeruginosa*	17 mm (200 µL/well) ^a^ 22 mm (200 µL/well) ^a^ 20 mm (200 µL/well) ^a^ 15 mm (200 µL/well) ^a^	[45]
	*S. aureus, E. coli* *P. aeruginosa, S. typhi, K. pneumoniae*	166.6 µg/mL 133.3 µg/mL	[46]
*P. atlantica*	*P. aeruginosa* PAO1	0.06 mg/mL ^b^ 0.50 mg/mL ^c^	[47]
	*E. coli*, *K. pneumoniae* *A*. *baumannii*	0.022 mg/mL, 0.044 mg/mL ^d^ 0.044 mg/mL, 0.044 mg/mL ^d^	[48]
*P. khinjuk*	*E. coli, K. pneumoniae, S. aureus* *S. mutans*, *E. faecalis* *S. mitis*	0.58 µg/mL 1.17 µg/mL 2.34 µg/mL	[49]
*P. chinensis*	*B. cereus*, *S. paratyphi*	25 mm (100 μg/disc) ^a^	[50]
*P. lentiscus*	*P*. *mirabilis*, *L*. *monocytogenes*	0.25% (*v*/*v*), 0.25% (*v*/*v*) ^d^	[51]
	*S. aureus* ATCC 29213 *B. cereus* *S. typhi* *E. coli* ATCC 25922 *C. albicans*	21.23 mm (10 µL/disc) ^a^ 15.18 mm (10 µL/disc) ^a^ 10.11 mm (10 µL/disc) ^a^ 16.07 mm (10 µL/disc) ^a^ 15.5 mm (10 µL/disc) ^a^	[52]
	*S. aureus. L. innocua*,	2.23 mg/mL, 4.46 mg/mL ^d^	[38]
	*M*. *luteus* *L. innocua* *E. coli* *F. oxysporum* f. sp. *albedinis*	70% ^e^; 75% ^e^ 60% ^e^ 60% ^e^; 50% ^e^ 54 ÷ 66% ^e^	[37]
	*S. aureus*, *P. aeruginosa*, *L. monocytogenes*	1.25 ÷ 2.5 µg/mL, 5 µg/mL ^d^	[53]
	*C. albicans* *V*. *cholerae* *S. aureus* MRSA	0.1 mg/mL, 5.0 mg/ml ^f^ 0.3 mg/mL, 0.5 mg/mL ^d^ 2.0 mg/mL, 3.0 mg/mL ^d^ 0.5 mg/mL, 1.0 mg/mL ^d^	[54]
	*K. pneumoniae* *Salmonella* spp. *C. albicans* *C. kefyr* *C. tropicalis* *S*. *cerevisiae* *A*. *niger*	0.6 mg/mL, 1.2 mg/mL ^d^ 1.2 mg/mL, 2.5 mg/mL ^d^ 1.2 mg/mL, 1.2 mg/mL ^f^ 2.5 mg/mL, 2.5 mg/mL ^f^ 5.0 mg/mL, 5.0 mg/mL ^f^ 5.0 mg/mL, 5.0 mg/mL ^f^ 2.5 mg/mL, 5.0 mg/mL ^f^	[39]

^a^ d—diameter of the inhibitory zone; ^b^ MBIC—minimum biofilm inhibitory concentration; ^c^ MIC (pyocyanin); ^d^ MBC—minimum bactericidal concentration; ^e^ RI—relative inhibition; ^f^ MFC—minimum fungicidal concentration.

**Table 4:** Method of Extraction, Bioactive Compounds and Potential Food Applications of Leaf Extracts from *Pistacia* Species.

*Pistacia* Species	Extraction Method	Main Bioactive Components	Food Applications
*P. vera*	UAE (50% EtOH) [9]	Male leaves: chlorophylls, limonene, and palmitic acid; female leaves: syringaldehyde and limonene	Preservation of vegetable oils, dairy products (cheese and butter), meat products, nuts, seeds, snacks
	UAE (50% EtOH) [11]		Bread additive, natural preservative
	MAE (70% EtOH) [8]	Male leaves: myricetin galloyl hexoside; female leaves: apigenin and quercetin-hexoside	Baking additive, prevents pathogen growth (e.g., *E. coli*, *S. aureus*) and microbial spoilage in processed and packaged foods
	Maceration (80% EtOH) [28]	Phloroglucinol, gallic acid, and vanillic acid	Preserve vegetable oils, dairy products, and snacks; functional foods
*P. atlantica*	Soxhlet (*n*-Hexane-**EtOAc**-**MeOH)** [30]	Gallic, ellagic, and 3,3′-dimethoxyellagic acids, as well as gallotannins	Natural antioxidants, functional ingredients, preservatives
	Hydrodistillation (H_2_O) [48]	Terpinen-4-ol and spathulenol	Natural food preservative, food safety enhancement, active packaging ingredient
	Maceration (MeOH) [29]	Gallic and cichoric acids	Food oxidative stability, functional ingredient, natural alternative to synthetic antioxidants (BHA and BHT), spoilage prevention, food quality
	Maceration (*n*-Hexane-EtOAc-**MeOH**) [47]	Myricetin-3-*O*-rutinoside, rutin, isoquercitrin, kaempferol-3-*O*-rutinoside	Biofilm formation inhibitor (*P. aeruginosa*), antimicrobial agent in food products, food preservative
*P. terebinthus*	Decoction (EtOAc, MeOH, H_2_O) [31]	EtOAc extract: phylloquinone, pheophytin B, and pheophytin A; MeOH and H_2_O extracts: procyanidin B, catechin, and epigallocatechin-3-*O*-gallate. Common for all extracts: quinic acid, gallic acid, rutin, luteolin	Antioxidant additive, functional food ingredient, natural preservative
*P. palaestina*	Maceration (96% EtOH) [32]	Quinic and gallic acids, as well as gallotannins	Natural antioxidant, functional ingredient, preservative
	Maceration (40% Acetone) [33]	Phenolic compounds	Natural antioxidant, functional ingredient, preservative
*P. integerrima*	Maceration (MeOH) [55]	Methyl benzoate, 1,5-dihydroxy-3-methylanthraquinone, gallic acid, betulin, 6,7,8-trimethoxycoumarin, rosmarinic acid, methyl rosmarinate, ole-an-12(13)-en-3β-hexadecanoate	Food safety enhancer, natural preservative, active ingredient in food packaging
*P. chinensis*	Maceration (EtOH) [34]	Phenolic compounds	Preservation of edible oil and fats, dairy products (butter, cheese), meat products, snacks, nuts and seeds, packaged and prepared foods
	Maceration (EtOAc, MeOH) [35]	Phenolic compounds	
	Maceration (80% MeOH) [50]	β-Sitosterol, lupeol, quercetin, myricetin, quercetin 3-*O*-α-rhamnoside, quercetin 3-*O*-β-glucoside, myricetin 3-*O*-α-rhamnoside, and myricetin 3-*O*-β-glucuronide	Preservation of meat and dairy products (e.g., *B. cereus, S. paratyphi)*
*P. khinjuk*	Maceration (H_2_O; Ag nanoparticles) [49]	Phenolic compounds	Active packaging ingredients, food preservatives, surface sanitization
*P. lentiscus*	UAE (Lactic acid) [42]	Quercetin derivatives	Antioxidant additive and chelating agent
	UAE (MeOH; H_2_O) [58]	Myricitrin and 2-hydroxy-1,8-cineole β-D-glucopyranoside	Fungal contamination control (e.g., *M. circinelloides*, *R. oryzae*), shelf-life extension
	MAE (70% EtOH) [41]	Myricetin glycosides, digalloylquinic acid	Natural antioxidant and preservative
	Soxhlet (CH_2_Cl_2_-**EtOAc**-EtOH-**MeOH)** [37]	Flavonoids	Food preservation, food safety and antispoilage agent (e.g., *M. luteus*, *L. innocua*, *E. coli*, *F. oxysporum*)
	Soxhlet (70% EtOH) [39]	3,5-Di-*O*-galloylquinic and gallic acids	Food additive with potential antidiabetic and anticoagulant benefits
	Soxhlet (EtOH abs.; H_2_O) [40]	Polyphenolics, flavonoids	Food stability, anti-inflammatory benefits, natural alternative to synthetic preservatives (BHT)
	Soxhlet (H_2_O) [39]	Galloyl quinic acids	Food preservation, poultry, and meat safety, antifungal agent
	scCO2 (CO_2_) [17]	Germacrene D, δ-cadinene, and α-pinene	Natural preservative, vascular health supplement
	Hydrodistillation (H_2_O) [51]	D-Limonene, 3-carene, and β-myrcene	Shelf-life extension (*C. albicans*, *S. aureus*, *E. coli*, *S. enterica*, *B. cereus*), food safety
	Hydrodistillation (H_2_O) [18]	β-Myrcene and α-pinene	Shelf-life extension, food safety improvement (*P. mirabilis*, *L. monocytogenes*, *P. aeruginosa*), antimicrobial enhancement
	Maceration (80% Acetone) [36]	Gallic acid, quercetin, and catechin	Natural alternative to synthetic antioxidants in various foods
	Maceration (Acetone) [59]	Pyrogallol and quercetin	Bacterial contamination control, shelf-life extension, food safety improvement (*S. aureus*, *P. aeruginosa*)
	Maceration (70% EtOH) [54]	Flavonoids, gallic and tannic acids	Natural antimicrobial agent, food preservative, functional food ingredient
	Maceration (MeOH, H_2_O) [53]	3,5-*O*-Digalloyl quinic acid, taxifolin, catechin hydrate	Natural antimicrobial agent, food preservative, active ingredient in food packaging
	Decoction (H_2_O) [38]	Flavonoids, volatiles	Food oxidative stability, active food packaging ingredient

## Data Availability

No new data were created or analyzed in this study. Data sharing is not applicable to this article.
